# Dynamic Emotional and Neural Responses to Music Depend on Performance Expression and Listener Experience

**DOI:** 10.1371/journal.pone.0013812

**Published:** 2010-12-16

**Authors:** Heather Chapin, Kelly Jantzen, J. A. Scott Kelso, Fred Steinberg, Edward Large

**Affiliations:** 1 Center for Complex Systems and Brain Sciences, Florida Atlantic University, Boca Raton, Florida, United States of America; 2 Department of Psychology, Western Washington University, Bellingham, Washington, United States of America; 3 Intelligent Systems Research Centre, University of Ulster, Magee Campus, Derry, North Ireland; 4 University MRI of Boca Raton, Boca Raton, Florida, United States of America; University of Barcelona, Spain

## Abstract

Apart from its natural relevance to cognition, music provides a window into the intimate relationships between production, perception, experience, and emotion. Here, emotional responses and neural activity were observed as they evolved together with stimulus parameters over several minutes. Participants listened to a skilled music performance that included the natural fluctuations in timing and sound intensity that musicians use to evoke emotional responses. A mechanical performance of the same piece served as a control. Before and after fMRI scanning, participants reported real-time emotional responses on a 2-dimensional rating scale (arousal and valence) as they listened to each performance. During fMRI scanning, participants listened without reporting emotional responses. Limbic and paralimbic brain areas responded to the expressive dynamics of human music performance, and both emotion and reward related activations during music listening were dependent upon musical training. Moreover, dynamic changes in timing predicted ratings of emotional arousal, as well as real-time changes in neural activity. BOLD signal changes correlated with expressive timing fluctuations in cortical and subcortical motor areas consistent with pulse perception, and in a network consistent with the human mirror neuron system. These findings show that expressive music performance evokes emotion and reward related neural activations, and that music's affective impact on the brains of listeners is altered by musical training. Our observations are consistent with the idea that music performance evokes an emotional response through a form of empathy that is based, at least in part, on the perception of movement and on violations of pulse-based temporal expectancies.

## Introduction

Although much research has focused on the ability of humans to detect and recognize emotional stimuli [1]–[3], we know less about affective responses that people experience in natural settings. Precise quantification of emotional reactions and their associated brain responses is challenging because emotional responses are often dynamic experiences that unfold on several timescales [4]. Emotional responses to identical stimuli vary among people, making it difficult to link specific stimulus parameters to individual responses. Moreover, measuring and imaging emotional responses simultaneously is problematic because explicit instructions to monitor and report emotional reactions can interfere with the affective responses one is attempting to measure [5]. Music has a number of key properties that make it an excellent model system for the study of emotion, addressing some of these issues. It is an ecologically valid stimulus that is used daily across cultures to communicate and modulate emotion. It is a dynamic stimulus whose parameters evolve over timescales ranging from fractions of a second to minutes. The existence of populations with and without music performance experience allows for the opportunity to explore the role of learning and experience in modifying the relationship between a stimulus and its associated emotional response. Here, we investigate the dynamics of emotional and neural responding to a natural music performance, focusing on the role of stimulus parameter dynamics and listener experience.

Previous neuroimaging work has revealed the involvement of several brain areas in emotional responses to music, focusing on contrasting musical attributes such as consonant/dissonant, pleasant/unpleasant, and happy/sad. Not surprisingly, areas associated with emotion processing and reward in general are also involved in emotional responding to music. Parahippocampus and precuneus activity were found to increase in response to increasing dissonance of short chord sequences [6], whereas increasing consonance was associated with activation of orbitofrontal and frontopolor cortices and subcallosal cingulate, a region implicated in emotion processing [7], [8]. Similarly, Koelsch, Fritz et al. [9] found unpleasant, compared to pleasant, excerpts activated parahippocampal gyrus as well as amygdala, and the temporal poles. Listening to pleasant, relative to unpleasant, music was associated with activation of insula, inferior frontal gyrus (IFG, including Brodmann Area (BA) 44), and the ventral striatum, a key structure in reward and addiction circuits [10]–[12]. Levitin and Menon [13] found blood oxygen level dependent (BOLD) response increases in non-musicians for normal versus temporally scrambled musical excerpts in IFG (BA 47), anterior cingulate, nucleus accumbens (part of the ventral striatum), brainstem, and posterior cerebellar vermis. These participants also showed increased BOLD responses in reward related brain areas for the normal excerpts. Another group of non-musicians rated the normal versions as more pleasant than the scrambled versions [14]. Similar emotion and reward related networks were found to be associated with increasing pleasurable chill ratings in response to listening to self-selected musical excerpts [15] and while listening to music rated as happy (versus sad) [16]. Thus, current research has identified emotion related limbic and paralimbic activations (e.g., amygdala, subcallosal gyrus, ventral anterior cingulate, and parahippocampal gyrus) and reward related activations (in ventral striatum) associated with affective responses to music. However, these approaches did not tackle the issue of how specific stimulus parameters may give rise to emotional responses.

Behavioral studies of emotion in music have focused on the role of specific musical parameters in communicating emotion [17]. In one approach, performers are asked to record short musical excerpts in a way that will convey basic emotions, such as anger, fear, or joy. Listeners then attempt to name the basic emotion that the performance was intended to convey. Regardless of musical training or cultural background, people are generally able to name the intended emotion, providing evidence that the expression of basic emotions (happiness, sadness, and fear) in music can be recognized universally [18], [19]. Moreover, listener judgments of intended emotion have been linked to specific musical features, including tempo, articulation, intensity, and timbre [20]–[23]. These studies have mostly used short excerpts and required participants to express their responses using single word labels. Thus, while listeners recognize intended emotions, it is possible that they do not actually experience emotional responses to music in such tasks [24]. Furthermore, short excerpts and discrete categories employed in many behavioral and neuroimaging studies do not capture dynamic aspects of musical emotion that unfold over larger time scales [24]–[26].

In order to explore dynamic affective responses of listeners to entire pieces of music, Schubert [24], [27], [28] developed a continuous response paradigm in which listeners report perceived emotion in real-time in a 2-dimensional emotion space [28], with emotional valence and arousal as the orthogonal dimensions [29]. In one study, Schubert [27] used four compositions to capture a wide range of musical feature variations and instructed participants to report “the emotion the music (is) trying to express.” Musical variables such as melodic contour, tempo, loudness, texture, and timbral sharpness, were shown to predict emotion ratings. Interestingly, tempo and loudness accounted for over 60% of the variance between musical pieces along the emotional arousal dimension. Musicians use fluctuations in timing and sound intensity within a performance to express structural interpretations and intensify emotional communication [30]. Other studies have considered dynamic fluctuations in tempo and sound intensity within individual performances. Koelsch et al. [31] showed that emotion-related physiological and neural responses to unexpected chords were greater when the chords were played in an expressive context. Bhatara et al. [32] created versions of music performances in which changes in timing and intensity were parametrically manipulated, and asked participants to rate the emotional expressivity. Emotion judgments monotonically increased with performance variability, and timing changes were found to explain more variance in reported emotional expressivity than sound intensity. Musically experienced listeners (with >6 yrs. of musical training) were more sensitive to performance expression than less experienced listeners. Sloboda and Lehmann [33] showed that in music performance, changes in tempo and sound intensity are correlated with one another, and with real-time ratings of emotional arousal. They also showed a systematic relationship between emotionality ratings, timing, and loudness when listeners rated their moment-to-moment level of perceived emotionality while listening to music performances.

These observations about expressive performance lead to several important questions. First, we asked whether listening to an expressive musical performance – compared to one that does not contain dynamic stimulus fluctuations – would lead to limbic and paralimbic activations in areas such as amygdala, parahippocampus, ventral anterior cingulate, and subcallosal gyrus, and perhaps to reward related activation in ventral striatum. We were also interested in understanding the relationship between feature variations, emotional responses, and neural activations within a temporally fluctuating musical performance. Based on previous studies [27], [32], [33], we expected to observe that real-time ratings of emotional arousal would correlate with fluctuations in tempo and sound intensity. Next, to illuminate the neural mechanisms of emotional responding to musical performance, we compared temporal performance fluctuations and reported emotional arousal with fluctuations in the BOLD signal. We hypothesized that tempo fluctuations would lead to violations of temporal expectancies [25], [34], [35] based on the perceived pulse [36]–[41], and that temporal expectancy violations would be associated with emotional responses. Activity in motor areas such as basal ganglia [42], [43], pre-SMA [42]–[44], SMA [42]–[45], and premotor cortex (PMC) [42]–[45] is present during rhythm perception, even in the absence of overt movement, and basal ganglia have been specifically linked to pulse perception [42], [46], [47]. Recently it has been shown that temporal unpredictability in the auditory domain is sufficient to produce amygdala activation in mice and humans [48]. Additionally, activity in IFG 47 has been linked to the perception of temporally coherent structure in music [13], and dorsal anterior cingulate cortex (dACC) has been associated with error detection in general [49] and could also be involved more specifically in temporal expectancy violations. Thus, it may be that activity in the motor areas related to rhythm and pulse perception, IFG 47, and dACC relate to temporal expectancy and violations of expectancy and that these violations may evoke emotion through activation of limbic areas such as the amygdala.

The current experiment focused on how performance expression influences the dynamic emotional responses to a musical stimulus that unfolds over a period of minutes. An expressive music performance, recorded by a skilled pianist, with natural variations in timing and sound intensity, was used to evoke emotion, and a mechanical performance was used to control for compositional aspects of the stimulus [35], [50], and for average values of tempo and sound intensity. To study emotional experience, “deep listeners” [51] were recruited. These participants reported listening to and enjoying classical music, but were not professional musicians and were not familiar with the piece used in the experiment. However, these non-expert listeners had varying degrees of musical experience and training, allowing us to address the role of moderate levels of musical experience (such as singing in a choir) in modulating emotional responses. Participants were asked to report their emotional responses to the music in real-time. Emotional responses were imaged separately to prevent self-report from interfering with experienced affect. This procedure was intended to increase the likelihood that participants would attend to and report their own emotional reactions to the music. Our specific hypotheses were that 1) listening to an expressive performance would result in limbic, paralimbic, and reward-related neural activations, 2) musical experience would affect emotion and reward-related activation, 3) real-time ratings of emotional arousal would correlate with fluctuations in tempo and sound intensity, and 4) tempo fluctuations would correlate with activation changes in cortical and subcortical motor areas related to the perception of musical pulse as well as brain areas related to error detection and expectancy violation.

## Methods

### Ethics Statement

Written informed consent was obtained for all participants. The study was approved by the IRB at Florida Atlantic University and was conducted in accordance with ethical guidelines for the protection of human subjects. Participants received research credits (between 1–3, depending on participation in the survey, behavioral, and/or fMRI portions of the study) to meet partial requirements for an undergraduate general psychology class.

### Participants

One hundred and twenty-five undergraduate psychology students completed a music response questionnaire (85 female and 40 male, aged 18–29 years, median  = 18 years) to determine eligibility for participation in the fMRI experiment. The questionnaire assessed musical background and personal responses to music. “Deep listeners” [51] were first identified as those who believed that their response to music and/or the role that music played in their lives was greater than that of the average person. Those who reported being moved by or having a strong emotional response to classical music were also identified. Twenty-seven participants qualified as deep listeners, and two additional participants were included who, while not deep listeners according to the criteria, reported having strong emotional responses evoked by classical music. Of these twenty-nine potential participants, twenty-one completed the fMRI study (14 female and 7 male, aged 18–21 years, median  = 18 years). Ten *experienced* participants were identified who had at least five years of music lessons or musical experience, such as playing in a band or singing in a choir. Two were undergraduate music majors. Of the remaining eleven *inexperienced* participants, eight reported no musical training or music-making experience whatsoever, two reported four years of experience playing music, and one reported one year of music lessons. One musically *inexperienced* male participant was not included in the fMRI analysis because of equipment failure. One *experienced* and one *inexperienced* participant (both female) were eliminated from the fMRI analysis because of excessive movement while in the scanner. Four additional participants were eliminated due to inconsistent behavioral report, or because their behavioral scores did not correlate with an *expressive* performance measure, as described in detail below. As a result, complete fMRI analysis was available for seven *experienced* (range of experience 6.5 to 18 years, mean  = 9.21 years, SD  = 4.04 years, 5 female) and seven *inexperienced* participants (range of experience 0 to 4 years, mean  = .71 years, SD  = 1.50 years, 4 female). Five of the seven *experienced* participants played piano (range of experience with piano 2–10 yrs, mean  = 6.1 years, SD  = 2.92 years), but none reported being familiar with the composition used in this experiment. The other two experienced participants played clarinet and flute (8 years and 7 years, respectively).

### Stimuli and Task

#### Sound Stimulus

Frédéric Chopin's Etude in E major, Op.10, No. 3 was performed by an undergraduate piano major (female, 22 years old) on a Kawai CA 950 digital piano and recorded in Cubase on a Macintosh G3 450 MHz computer [35]. The performer was asked to rehearse and play the piece as she would in a performance (*expressive* performance, see supporting materials for the audio file S1). A *mechanical* performance was synthesized on the computer by changing the onset time and duration of each note to precisely match that of the musical notation. The MIDI (Musical Instrument Digital Interface) onset velocity (key pressure) of each note (correlating with sound level) was set to 64 (range 0–128), and pedal information was eliminated. Despite the omission of pedal information, the performance sounded natural (see supporting materials for the audio file S2.) Mean tempo of the *mechanical* performance was adjusted to equal the mean tempo of the *expressive* performance, making the duration of the *mechanical* performance equal to the *expressive* (3 minutes and 36 seconds). Finally, the MIDI files were synthesized through the Kawai CA 950 digital piano to create audio files, and the root mean square (RMS) amplitude of the *mechanical* performance was adjusted to equal the mean RMS amplitude of the *expressive* performance. Therefore, the *mechanical* performance did not include expressive changes in tempo (rubato) or sound level (dynamics) and the *expressive* performance varied in both tempo and sound intensity about a mean common to both performances.

#### Behavioral Task

**Figure 1:**
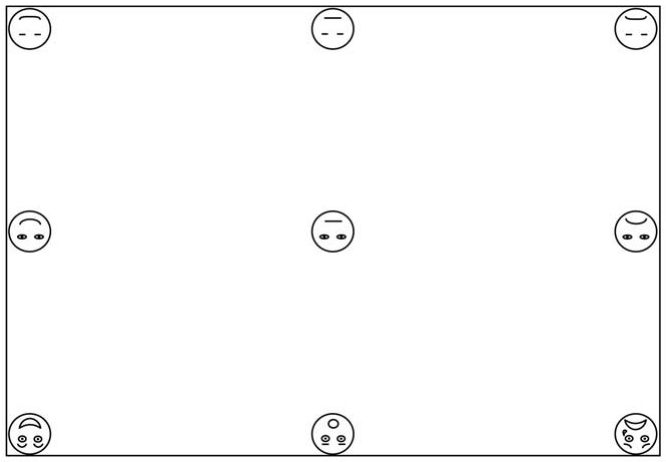
2-Dimensional emotion response space. Valence is represented on the horizontal dimension and arousal is represented on the vertical dimension.

Sound stimuli were presented using MaxMSP 4.2.1 software running on a PowerBook G4. A 2-dimensional emotion response space, adapted from Schubert [52], was presented visually so that participants could report emotional responses to the performances in real time (Figure 1). The two dimensions were emotional arousal (vertical dimension on a scale from 0 to 10) and emotional valence (horizontal dimension on a scale from −10 to 10). Participants were instructed to move the mouse cursor to the position in the emotion response space that best matched their emotional responses to the music being played. They were instructed to “tell us about (their) own emotional experience” rather than the emotion they thought the piece was trying to express. They were told that higher arousal values corresponded to feeling an emotion more intensely, positive valence values corresponded to positive emotions (like happiness or excitement), and negative valence values corresponded to negative emotions (like sadness or anger). The position of the cursor for all participants started at zero arousal, zero valence (bottom middle point in the response space). The software recorded cursor position automatically during music playback with an average sampling period of 135 ms. Participants performed this task immediately before entering the scanner and after scanning, but not during fMRI acquisition. Two behavioral sessions were conducted to test reliability of reported emotional responses. It was assumed that if participants' reported emotional responses were reliable over time, similar emotional responses would be experienced in the scanner, allowing for correlations between behavioral and physiological data. Performances were presented in counterbalanced order across the two sessions.

#### fMRI Recording

A custom Visual Basic 5 program running on a Dell Optiplex GX260 was used to play sound stimuli which were presented to participants using custom noise-attenuating headphones (Avotec, Inc). They were instructed to lie motionless in the scanner with eyes closed and listen attentively to the music without actively monitoring or reporting their emotional response. During the rest period, participants were instructed to rest quietly with eyes closed and wait for the music to begin again.

### Magnetic Resonance Imaging

Changes in blood oxygenation (BOLD response) were measured using echo planar imaging on a 3.0 T Signa Scanner equipped with real time fMRI capabilities (General Electric Medical Systems, Milwaukee, WI). Echo-planar images were collected using a single shot, gradient-echo, echo planar pulse sequence (field of view (FOV)  = 24 cm, echo time (TE)  = 35 ms, flip angle (FA)  = 90°, in plane resolution  = 64×64). All images were collected using a sparse temporal sampling technique with a repetition time (TR) of 12 seconds. Adequate coverage of the brain was achieved by collecting thirty interleaved 4 mm axial slices with no spacing between (voxel size  = 3.75×3.75×4 mm). Immediately following the functional imaging, high resolution anatomical spoiled gradient-recalled at steady state (SPGR) images (4 mm thick, no spacing, number of excitations  = 2, TE  =  in phase, TR  = 325 ms, FA  = 90°, in plane resolution 256×256, bandwidth = 31.25) were collected at the same slice locations as the functional images.

#### Acquisition

**Figure 2:**
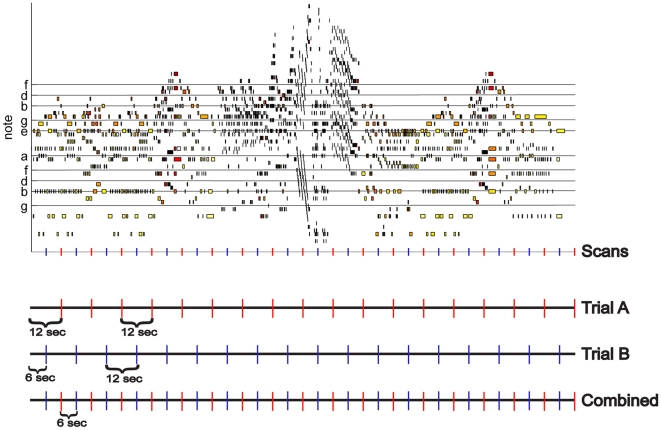
Piano roll notation and fMRI scan times. Piano roll notates Chopin's Etude in E major, opus 10, no .3.

A sparse temporal sampling technique was used in the scanner to increase the signal response from baseline (which was silence) and to avoid nonlinear interaction of the scanner sound with the auditory stimulus [53]. There were a total of two trials. Within each trial, there was one minute of rest between the two stimuli and after the last stimulus presentation. One trial started with twelve seconds of rest, followed by the *expressive* performance and then the *mechanical* performance. The other trial started with six seconds of rest, followed by the *mechanical* and *expressive* performances. The variable amount of time in the first rest period enabled imaging of 36 unique time points over the two trials. Thus, when combined, the scans yielded an effective repetition time (TR) of 6 seconds (Figure 2). The total number of scans acquired for each participant over both trials was 96 (18 scans per performance per trial (36 scans total for each performance) +24 rest scans across trials). Trial order was randomized across participants. In summary, participants performed the real-time emotional rating task immediately prior to scanning, then were scanned without reporting emotional responses for two trials of each performance, and finally performed the real-time emotional rating task immediately after scanning.

### Data Analysis

#### Behavioral measures

Data analysis was performed using Matlab 7.2.0 (The MathWorks, Inc.) running on an Apple G5. First, the performance was matched to its score using a custom dynamic programming algorithm [54], [55]. Chords were grouped by the same dynamic programming algorithm, and onset time of a chord was defined to be equal to the average of component note onset times. This procedure enabled the identification of timing fluctuations. Beat times were extracted as the times of performed events that matched notated events occurring on sixteenth-note level beats. Sixteenth-note level beats to which no event corresponded were interpolated using local tempo. Inter-beat intervals (IBIs, in seconds) were calculated by subtracting successive beat times, and a tempo curve in beats per minute (bpm) was constructed according to the formula (local tempo  = 60/IBI). Each value in the tempo curve was divided by 4 because calculations had been made at the sixteenth note level (four sixteenth notes per quarter note; for details see [35]). One measure provided by MIDI is the velocity at which the key is struck, which is related to sound intensity [56]. A velocity curve, showing how sound intensity changed over the piece, was created by averaging the velocities of notes in chords. These procedures resulted in tempo and velocity curves that were sampled at event onset times, i.e. at unequal sampling intervals. Therefore, the tempo and velocity curves were resampled at equal intervals (10 ms; i.e. 100 Hz). Next, the frequency content of the tempo curve was measured using a fast Fourier transform, and the Nyquist frequency was determined to be approximately 0.5 Hz. Finally, to prevent spuriously high correlations with emotion rating time-series (discussed next) due to over-sampling, the time-series data were low-pass filtered and down-sampled at 2-second intervals (0.5 Hz).

Arousal and valence time-series were decimated to 0.5 Hz to match the sampling rate of the tempo curve. Then they were correlated across trials at an optimal time lag to test for reliability. For participants whose ratings were reliable, means of first and second trial rating curves were calculated for use in subsequent analyses. Next, optimal lag times (12-second maximum) were calculated for correlations between the behavioral data and the tempo curve. Finally, mean arousal and valence ratings, each advanced by the optimal lag time, were correlated with the tempo curve of the *expressive* performance.

#### fMRI

Data analysis and display were performed using AFNI [57], [58] and image registration was conducted using FSL (Analysis Group, FMRIB, Oxford, UK), both running on an Apple G5. Functional data sets were corrected for motion, and mean scaling of intensity was globally normalized. To model the BOLD response to the presence of music, a hemodynamic response function (HRF) was convolved with a binary vector representing the off/on timing of each condition (performance  =  on, silence  =  off, with each performance modeled separately). Each participant's head motion information, obtained during volume registration, was added to the baseline model to account for the variance due to head motion in all of the comparisons. A general linear approach, as implemented in AFNI, was used to determine the contribution of the model to the data from each voxel. Functional images were then coregistered and transformed into Talairach & Tournoux [59] coordinates.

A separate second-level analysis was used to compare activations across participants. A mixed-design 2-way ANOVA with the factors performance type (*expressive vs. mechanical*) and musical experience (*experienced* vs. *inexperienced*) was conducted on the beta weights from each voxel. To correct for multiple comparisons, a Monte Carlo simulation was conducted to determine the random distribution of voxel cluster sizes for a given threshold [60]. A two-tailed alpha level of *p*<.02 was achieved through the combination of a per voxel threshold of *p*<.05 and a cluster size of 10 contiguous voxels (640 mm).

Additional analyses were performed to determine the contribution of dynamic fluctuations in stimulus tempo and reported emotional arousal to BOLD activity. The tempo curve and the time-advanced mean arousal ratings were filtered with a hemodynamic response function (HRF) and were resampled at six-second intervals to match the sampling rate of the fMRI data. HRF filtered emotion ratings were then normalized about 0 by dividing each time-series by its mean and subtracting 1. The HRF filtered tempo curve for the *expressive* performance and the HRF filtered arousal ratings of each participant were regressed against the BOLD time series. The *expressive* and *mechanical* performance models used in the first analysis were added to the baseline model to account for tonic changes in BOLD intensity associated with listening to the music. Head motion parameters were also included as part of the baseline model. Group analysis was conducted by submitting individual beta weights to between subject *t*-tests. Again, a Monte Carlo simulation was conducted to correct for multiple comparisons and an alpha level of *p*<0.02 was achieved with a per voxel threshold of *p*<0.02 and a cluster size of 12 contiguous voxels (768 mm).

## Results

### Behavioral measures

**Figure 3:**
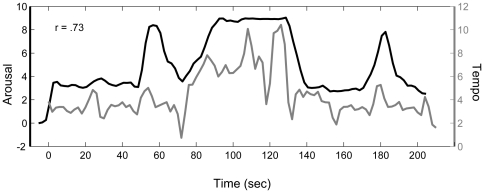
Correlation between tempo and mean arousal for the expressive performance for one participant. For this participant, the optimal lag time was −5.4 seconds and time shifted arousal ratings are shown. (r = .73, p<.0001)

For the *expressive* performance, the patterns of emotional arousal ratings were significantly positively correlated across the two trials for nineteen out of twenty-one participants (mean *r* = .62, *SD*  = .23, *p*<.05). Emotional valence ratings were less reliable across trials, but still significantly positively correlated in eighteen out of twenty-one participants (mean *r* = .46, *SD*  = .22, *p*<.05). Given the reliability of emotion ratings across trials, mean arousal and valence ratings were calculated across the two trials for each participant for comparison with dynamic measures of the music performance. Mean arousal and valence ratings were positively correlated in six participants, negatively correlated in nine participants, and uncorrelated in six participants. Therefore, arousal and valence ratings did not have a reliable relationship across subjects. See Table 1 for a summary of the behavioral results. Regression analysis revealed that BOLD filtered tempo and velocity (loudness) curves (as described in the Methods section) for the *expressive* performance were highly correlated (*r* = .87, *p*<.05). For this reason, and because previous findings suggest that tempo fluctuation accounts for more variance in emotionality ratings than loudness changes in performed music [32], subsequent analyses focused on tempo changes. Arousal ratings were significantly positively correlated with the tempo curve in nineteen out of twenty-one participants at their optimal time lag (mean of significant correlations *r* = .50, *SD*  = .20, *p*<.05, mean lag 7.2 seconds). Figure 3 illustrates the relationship between one participant's mean emotional arousal ratings and the tempo curve of the *expressive* performance. Linear regression was used to test the pairwise correlations between participants' mean arousal rating time series. All but two (99.5%) of the pairs were significantly correlated at the p<0.05 level (mean r = .62). Thus, arousal measures were highly reliable across participants. Mean valence ratings were not as reliable across participants (mean r = .32, 64.3% of pairs were significantly correlated at p<0.05). Emotional arousal correlations with the tempo curve of the expressive performance did not differ significantly between *experienced* and *inexperienced* participants (paired *t*(6) = .97, *p* = .18). Emotional valence ratings were significantly negatively correlated with the tempo curve of the *expressive* performance in sixteen out of twenty-one participants and significantly positively correlated in four out of twenty-one participants (mean *r* = −.25, *SD*  = .32, p<.05). Because of the stronger and more reliable relationship between tempo and arousal, arousal was focused on in subsequent analyses. Additionally, only participants whose arousal ratings correlated significantly with tempo were included in further analyses (eliminating one *experienced* and one *inexperienced* participant). In summary, tempo fluctuation in the *expressive* performance predicted emotional arousal ratings for both *experienced* and *inexperienced* participants.

**Table 1:** Behavioral correlations based on reported emotional responses, *p<.05.

Subject	Arousal (Trial 1/Trial 2)		Valence (Trial 1/Trial 2)		Arousal – Valence (Mean of Trials)		Arousal – Tempo (Mean of Trials)	
Exp1	0.77	*	0.30	*	−0.56	*	0.54	*
Exp2	0.70	*	0.50	*	0.04	*	0.51	*
Exp3	0.65	*	0.10		0.26		0.71	*
Exp4	0.62	*	0.41	*	−0.87	*	−0.10	
Exp5	0.80	*	0.65	*	0.14	*	0.41	*
Exp6	0.53	*	0.48	*	−0.21		0.66	*
Exp7	0.64	*	0.64	*	−0.57	*	0.71	*
Exp8	0.04		0.51	*	−0.21		0.33	*
Exp9	0.63	*	0.47	*	0.64	*	0.59	*
Exp10	0.87	*	0.50	*	0.78	*	0.73	*
Inexp1	0.75	*	0.83	*	0.24	*	0.55	*
Inexp2	0.76	*	0.80	*	0.54		0.49	*
Inexp3	0.52	*	0.16		−0.16	*	0.43	*
Inexp4	0.75	*	0.59	*	−0.71	*	0.63	*
Inexp5	0.78	*	0.68	*	−0.80	*	0.75	*
Inexp6	0.57	*	0.01	*	−0.08	*	0.44	*
Inexp7	0.71	*	0.63	*	−0.67	*	0.18	
Inexp8	0.74	*	0.52	*	−0.31		0.54	*
Inexp9	0.73	*	0.35	*	0.19		0.62	*
Inexp10	−0.07		0.20	*	0.07	*	0.47	*
Inexp11	0.46	*	0.35	*	−0.15	*	0.34	*
Mean	0.62		0.46		−0.11		0.50	
St. Dev.	0.23		0.22		0.47		0.20	

### fMRI

#### ANOVA

**Figure 4:**
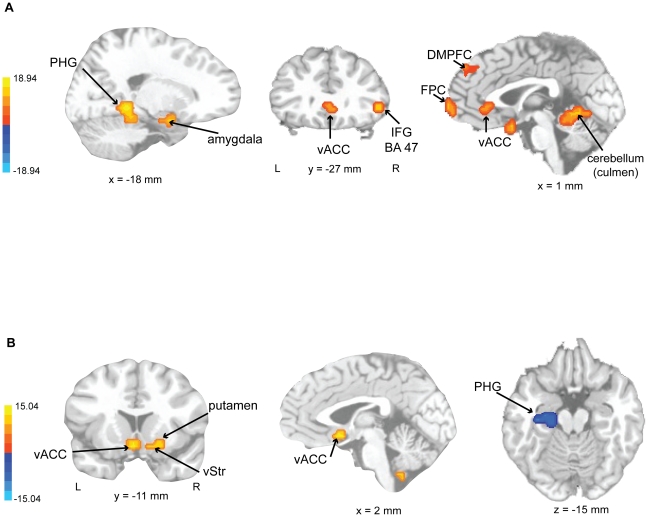
fMRI ANOVA results. Brain activations (F-maps) showing a significant main effect of a) performance type (F (1,24) > 7.19, corrected p < .02), SCG  =  subcallosal gyrus, PHG  =  parahippocampal gyrus, vACC  =  ventral anterior cingulate, FPC  =  frontopolor cortex, DMPFC  =  dorsal medial prefrontal cortex; and b) main effect of musical experience, BG  =  basal ganglia, vStri  =  ventral striatum.

Table 2 and Figure 4 present summaries of the fMRI ANOVA results. A main effect of performance type (*expressive* versus *mechanical*) was the result of an increased BOLD signal for the *expressive* performance in right posterior parahippocampal gyrus (extending into amygdala and hippocampus), fusiform gyrus, inferior parietal lobule (BA 40), IFG (BA 47), anterior parahippocampal gyrus, bilateral ventral anterior cingulate (predominately right lateralized), left medial prefrontal cortex (BA 10, frontopolor area), right dorsal medial prefrontal cortex (BA 8) and precuneus (BA 7). An increased BOLD response for the *mechanical* performance was observed only in the supramarginal gyrus (BA 40).

**Table 2:** Brain activations (ANOVA results) showing a significant main effect of performance type and musical experience (F (1,24) > 7.19, corrected *p* < .02).

	REGION (cluster peak)	BA	Cluster includes	X	Y	Z	Volume (mm^3^)	F-values
**Main Effect: Expressive vs. Mechanical**								
R	parahippocampus	30	bilateral culmen and vermis	18	−41	−4	8256	18.94
R	fusiform gyrus		declive (19, 37)	42	−57	−12	2752	10.96
R	inferior parietal	40	superior parietal (7)	38	−37	48	2304	10.73
R	inferior frontal	47		46	27	0	1088	9.63
R	parahippocampus	34	amygdala, hippocampus, subcallosal (34)	14	−1	−16	1024	10.25
L	medial frontal (frontopolar)	10		−2	67	4	1024	9.18
R	ventral anterior cingulate	24,32	bilateral (24)	6	35	−4	1024	8.16
L	precuneus	7		−30	−49	48	1024	8.3
L	supramarginal (Decrease)		inferior parietal (40)	−58	−45	28	768	−10.34
R	medial frontal	8		2	51	40	768	8.39
L	superior frontal	8		−14	43	48	640	8.3
**Main Effect: Experienced vs. Inexperienced**								
R	ventral striatum		lentiform, putamen, subcallosal	14	11	−8	1664	7.91
L	parahippocampus (Decrease)	35	hippocampus	−22	−21	−16	1088	−8.16
L	ventral anterior cingulate		(10)	−14	39	4	960	13.82
L	ventral anterior cingulate	25	subcallosal	−2	11	−8	704	15.04

A main effect of musical experience was found with greater BOLD activity for *experienced* compared to *inexperienced* listeners occurring in right ventral striatum (extending into lentiform nucleus, putamen, and subcallosal gyrus) and left ventral anterior cingulate. *Inexperienced* listeners had greater BOLD activity than *experienced* listeners in the left anterior parahippocampal/hippocampal gyrus (see Figure 4b).

**Figure 5:**
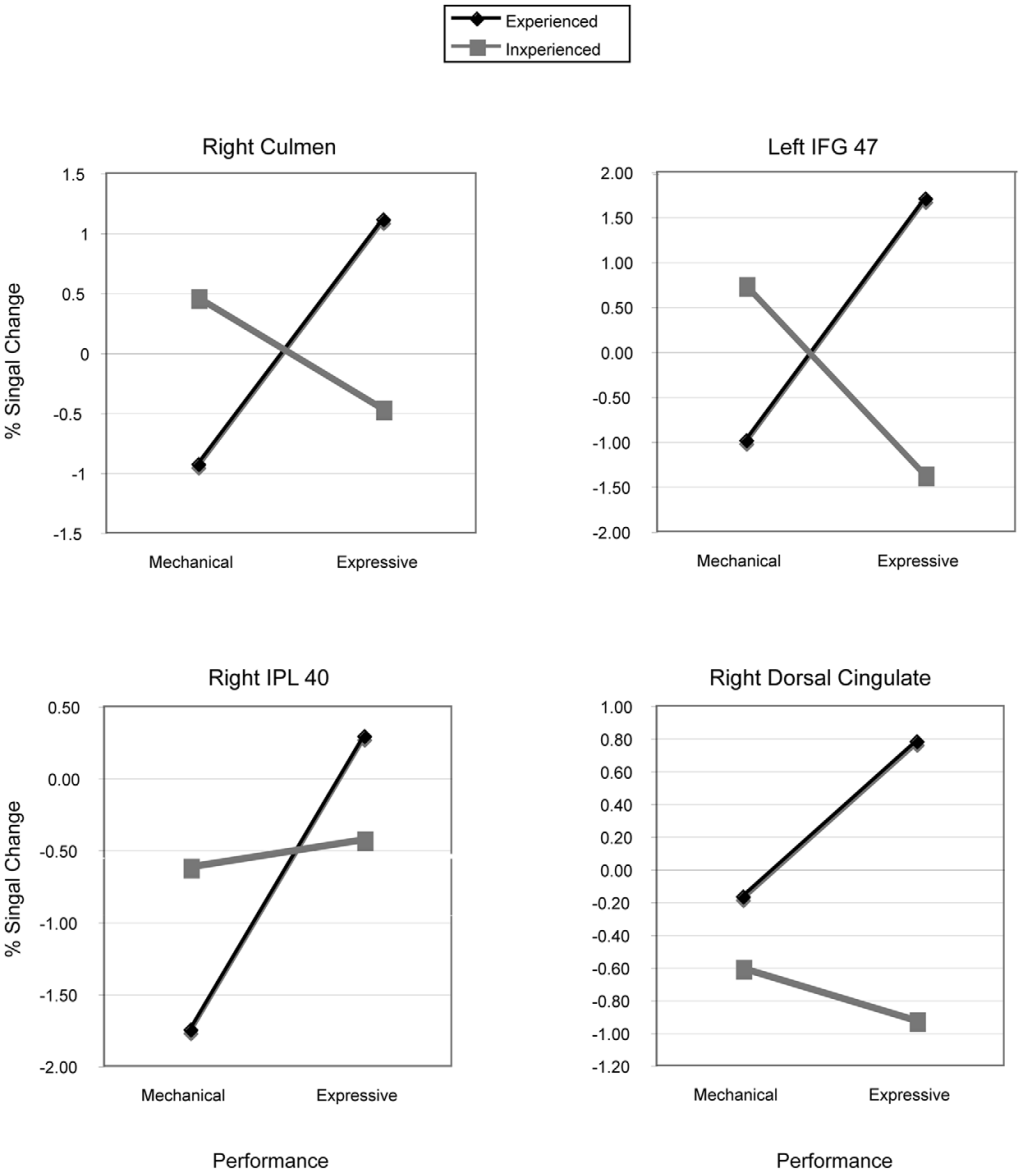
Peak voxels showing a significant interaction between performance type and experience. IPL  =  inferior parietal lobe.

Several brain regions showed a significant interaction of performance type with musical experience (see Figure 5). In the left cerebellum (culmen), left IFG (BA 47), right inferior parietal lobe (BA 40), and right dorsal cingulate (near pre-SMA), *experienced* participants showed a greater percent signal change for the *expressive* than the *mechanical* performance whereas *inexperienced* listeners showed the opposite response.

#### Tempo

**Figure 6:**
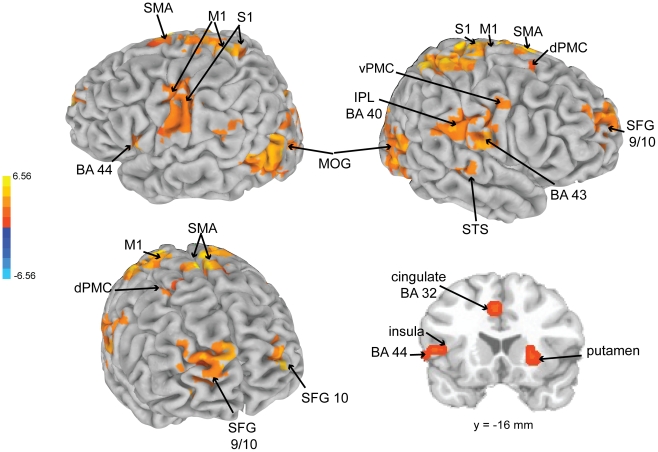
Brain activations as a function of the tempo of the expressive performance. SFG  =  superior frontal gyrus, dPMC  =  dorsal PMC, SMA  =  supplementary motor area, S1  =  primary somatosensory cortex, M1  =  primary motor cortex, vPMC  =  ventral PMC, STS  =  superior sulcus.

BOLD signal changes in a number of areas showed significant positive correlations with the tempo curve of the *expressive* performance (see Table 3 and Figure 6). They included right lingual gyrus, PMC, left primary motor and somatosensory cortex (extending into inferior parietal lobe BA 40), right postcentral gyrus (BA 43, extending into primary motor, PMC, and inferior parietal lobe BA 40), bilateral dorsal lateral prefrontal cortex (right BA 9/10 extending into ventral anterior cingulate, left BA 10), right lentiform nucleus and putamen, left insula, BA 44, and right secondary auditory cortex (BA 21 and 22).

**Table 3:** Brain activations as a function of expressive tempo and reported emotional arousal, corrected *p*<0.02, df = 12.

	REGION (cluster center)	BA	Cluster includes	X	Y	Z	Volume (mm^3^)	T-values
	**Tempo**							
R	lingual gyrus		cuneus (17, 18), bilateral cuneus (17), L culmen, vermis and declive, L posterior cingulate, L middle occipital, inferior occipital	6	−89	4	33088	2.98
R	medial frontal, SMA	6	SFG (6), precentral (6), bilateral precentral (4), postcentral (3, 5), bilateral cingluate	2	−13	68	28800	2.68
R	lingual gyrus	18	middle occipital (18, 19), inferior occipital, fusiform gyrus (19, 37), MTG (37)	18	−81	−4	13504	3.42
L	postcentral	3	precentral (4, 6), inferior parietal, insula (13)	−46	−17	48	11840	2.76
R	postcentral	43	precentral (4, 6), insula (13), inferior parietal	58	−9	16	10496	3.50
R	superior frontal	9	middle frontal (10), ventral anterior cingulate	10	59	32	6784	2.97
L	insula	13	pulvinar of thalamus	−30	−29	16	1920	3.07
L	precentral		insula (13), precentral (44)	−50	7	8	1536	3.43
L	superior frontal	10	middle frontal (10)	−22	59	16	1408	2.61
R	superior temporal	21	(22)	62	−21	0	1216	2.99
R	insula	13		38	−5	16	1024	3.65
R	lentiform nucleus, putamen			22	15	4	832	3.49
L	insula	13		−42	−13	4	768	2.73
	**Emotional Arousal**							
L	cuneus	19	(18)	−14	-89	24	1280	3.01
R	middle occipital		(19)	30	−85	16	1088	2.80
	**Emotional Arousal: Experienced vs. Inexperienced**							
L	middle frontal		precentral (6, 4), IFG (44, 45, 9)	−46	7	44	3840	2.91
R	cingulate	24	R anterior cingulate (32), bilateral to L (32, 24)	2	11	32	3840	3.15
L	inferior parietal		supramarginal, insula (13)	−46	−41	28	2880	3.04
L	insula	13	STG (22, 41), MTG (22)	−42	−25	16	2240	3.09
L	inferior frontal	47	(45), precentral (44)	−42	23	0	2176	2.82
R	middle temporal	21	STG (22, 39)	62	−49	8	1472	3.34
R	superior frontal/medial frontal	6	pre-SMA	2	3	64	1344	3.39
R	inferior parietal	40	insula	58	−29	36	1152	3.00
R	middle frontal	9	cingulate	54	19	28	1152	3.02

#### Emotional Arousal

**Figure 7:**
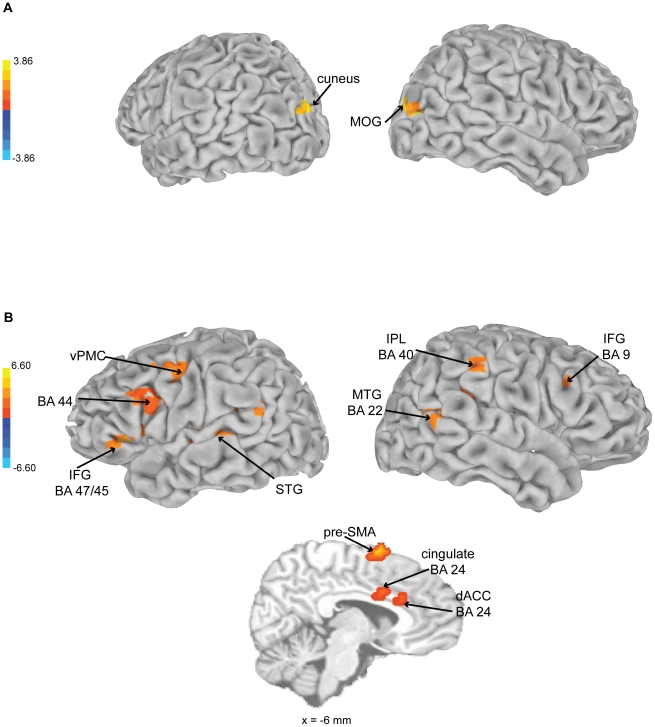
Brain activations as a function of emotional arousal ratings. Brain activations shown as a function of a) emotional arousal ratings in response to the *expressive* performance for all participants, MOC  =  middle occipital gyrus, dACC  =  dorsal anterior cingulate, MFG  =  middle frontal gyrus, MedFG  =  medial frontal gyrus; and b) emotional arousal ratings in response to the *expressive* performance for *experienced* versus *inexperienced* participants.

A positive correlation between BOLD signal changes and real-time reports of emotional arousal was found in left cuneus and right middle occipital gyrus. *Experienced* relative to *inexperienced* listeners showed positive correlations between BOLD signal changes and emotional arousal ratings in left premotor cortex (extending into IFG BA 44 and 45), right cingulate gyrus (including dorsal anterior cingulate), left inferior parietal lobe, insula, IFG (BA 47), right secondary auditory cortex, pre-SMA, inferior parietal lobe (BA 40) and lateral prefrontal gyrus (see Figure 7 and Table 3). The differences seen between *experienced* and *inexperienced* participants in the aforementioned contrast were due to significant positive correlations between the *experienced* participants' brain data and their emotional arousal ratings rather than due to significant negative correlations between *inexperienced* participants' brain data and their emotional arousal ratings.

## Discussion

We asked whether the temporal dynamics of an expressive musical performance would lead to emotion and reward related neural activations, and whether musical experience would modulate these responses. fMRI scanning revealed activation of emotion-related structures for the expressive performance including parahippocampal gyrus, amygdala, ventral anterior cingulate, and dorsal medial prefrontal cortex. The experimental design controlled for compositional features such as melody, harmony, and rhythm, and for performance parameter means (tempo and sound intensity), therefore we conclude that the temporal dynamics of timing and sound intensity were responsible for the increased limbic and paralimbic activations. Moreover, the expressive temporal dynamics of the performed music predicted listener ratings of emotional arousal as they evolved over time. Musically experienced listeners were found to have increased activation (compared to inexperienced listeners) in ventral striatum, basal ganglia (lentiform nucleus, putamen), subcallosal gyrus, and left ventral anterior cingulate. The experienced listeners were not professional musicians; they had moderate levels of experience performing music. Yet they exhibited enhanced neural responses related to emotion and reward when compared to inexperienced participants. Musical involvement may lead to enhanced neural responses, or alternatively, enhanced emotional responding to music may lead one to seek out musical activities. Nevertheless, these results provide evidence that temporal dynamics of expressive rhythmic performance increase emotion-related neural activations, and that musically experienced listeners are more sensitive in this regard.

In accord with previous work linking amygdala response to temporal unpredictability in the auditory domain [48], it was found that the right amygdala was more active for all participants when listening to the expressive performance. It should be noted, however, that the amygdala is also activated by rising sound intensity in humans [61] and that changes in tempo and sound intensity are highly correlated in performed music in general [62], and in this performance in particular. It remains to be demonstrated whether tempo or intensity changes alone could cause the effect we have demonstrated here, and it is possible that the interaction between tempo and sound intensity is required for the emotional response of listeners. Furthermore, it is not clear from the present experiment whether tempo or intensity changes presented without pitch information would be sufficient for the activation of emotion-related structures. It may be that emotional arousal would be affected by expressive changes embedded in a purely rhythmic context, or alternatively, the presence of a full melodic and harmonic musical context may be required to engage the neural activations we have observed here. Future work is needed to decouple the emotion-related effects of timing and sound intensity changes from one another and from a musical composition.

To address the question of how expressive performance leads to increased emotion-related activity, tempo fluctuations and real-time ratings of emotional arousal were compared with dynamic changes in BOLD activation. Here, our observations were consistent with an expectancy theory of emotional responding to music [25], [34], [63]. The temporal unpredictability of the performance used in this study was investigated in an earlier pulse tapping study [35], which showed that listeners can predict the pulse of the expressively timed musical performance, though not as accurately as when tapping the pulse of the mechanical performance. The perception of pulse is thought to underlie the development of temporal expectancies [36], [41]. In this study, we observed that activation of motor-related areas, such as pre-SMA, SMA, PMC, primary motor, basal ganglia (lentiform nucleus, putamen), cerebellum, and thalamus correlated with the tempo of the expressive performance. Activation of these motor regions has previously been reported during rhythm perception [64], [65]. Basal ganglia activation has been specifically linked to pulse perception [42], [46], [47] and has been shown to be involved in emotion processing in general [7]. Additionally, in this study, dACC activity correlated with emotional arousal in the experienced participants. Thus, consistent with its involvement in error detection and correction [49], the dorsal portion of the anterior cingulate may play a role in the detection and correction of temporal expectancy violations in music. Moreover, activity in IFG (BA 47) was associated with listening to the expressive performance overall, and more so for experienced participants. Previous work has linked IFG 47 (along with insula activity) to the perception of temporal structure in music [13]. Along with the previously mentioned experiment conducted by Bhatara et al. [32], these results suggest that musically experienced listeners may be more aware of and respond more to tempo fluctuations in performed music. Based on their involvement in the perception of pulse and temporal structure in general, it could be that motor areas (especially basal ganglia) and BA 47 contribute to the development of temporal expectancies, and that their activity, combined with dACC, contributes to the experience of temporal expectancy violations.

An unexpected finding of this study was that, for all participants, the tempo fluctuations of the expressive performance correlated with dynamic activation changes in brain regions that are consistent with the human mirror neuron system [66], including bilateral BA 44/45, superior temporal sulcus, ventral PMC, and inferior parietal cortex, along with other motor-related areas and with insula (see file S3 in the supporting materials for a visualization of the effect of tempo on these areas). Rizzolatti and Craighero [66] theorized that the mirror neuron system provides a mechanism for mapping the actions and intentions of others onto our own motor system as a means of action understanding. Mirror neuron activity has been reported when listening to common action sounds [67] and to previously learned music [68]. Molnar-Szakacs & Overy [69] hypothesized that the mirror neuron system provides a mechanism by which listeners may experience music empathically rather than by cognitively interpreting an internal representation. In other words, the communication of emotion during music listening is an empathic process in which a listener *feels* the emotion communicated by the performer. A mechanism for this action could involve engagement of the mirror neuron system and its interaction with the limbic system via the insula [70]. Tempo and sound intensity, parameters that are manipulated through emotionally charged movement, are two properties of music that may convey emotion from performer to listener through a process of motor resonance. Thus, perception of motion in music [71] may occur through activation of mirror neuron and motor systems and lead to emotional responses through interactions of the mirror neuron network and limbic system taking place via the insula [70]. In conjunction with the observation of motor networks involved in pulse perception, the current observations suggest the possibility that listeners perceive motion in the dynamic fluctuations of music performance, and this results in a form of empathic motor resonance leading to emotional responses [69].

It has been shown that those with musical training listen to music differently [72], exhibit different activation patterns during music perception [73]–[76], show enhanced processing of affective vocal sounds [77], and even show differences in brain anatomy [78], [79]. Also, expertise in a particular type of movement, such as dance, has been shown to alter the way mirror neurons respond during observation [80], demonstrating that experience is an important factor in determining whether perceptions will resonate with one's own motor repertoire. In the current study, mirror neuron regions were correlated with dynamic ratings of emotional arousal only in the experienced listeners, who also showed increased activation in emotion and reward-related areas. Thus, the pathways mediating emotional responses based on temporal fluctuations may differ with musical experience. It may be that all listeners perceive motion arising from changes in tempo through activation of mirror neuron and motor systems. However, it is possible that mirror neuron activity only influences emotional responses in listeners with explicit experience conveying emotion through music performance. Therefore, network interactions between the mirror neuron system, insula, and limbic system may be more readily engaged in those with musical experience. Such individuals have the experience of conveying emotion through music performance and may have a more developed mapping between musical structure, motor experience, and emotion.

Increased sensitivity to expressive performance parameters may help explain why experienced participants showed enhanced responses in areas related to emotion, memory, and reward. Overall, listening to the expressive performance increased activity in parahippocampal gyrus, hippocampus, medial prefrontal cortex (mPFC), and ventral anterior cingulate (vACC) for all participants. Experienced participants showed a greater response than inexperienced listeners in the ventral striatum and ventral anterior cingulate while listening to music in general. The ventral striatum (including nucleus accumbens) has been associated with the rewarding property of music [9], [14]–[16]. The vACC has been shown to be related to emotional processing in general [7], [49] and more specifically in music [15], [16]. The mPFC, specifically BA 10, has been implicated in emotion processing and attending to one's own emotional state [81]. Parahippocampal gyrus activity has been shown in previous studies investigating emotional responses to music [6], [9], [16] and has been linked to understanding social contextual cues [82]. These results suggest that expressive music performance activates emotion related structures in listeners and that even a moderate level of musical experience enhances this emotion associated activation and increases the rewarding aspect of music listening, perhaps through the engagement of the mirror neuron system and its interactions with the limbic system via the insula. Familiarity with a specific musical piece does not seem to be necessary to evoke this response.

The journey from listening to feeling involves a dynamic interplay between several neural systems [83]. Auditory and attentional systems (which are both modulated by previous experience, expectancy, and initial emotional conditions) must attend to and integrate sensory information into temporally and harmonically coherent structures. The dynamic integration of incoming musical stimuli with ongoing neural processes––which perhaps reflect previously developed expectancy––will result in the formation, strengthening, and maintenance of expectancies if the stimulus properties match the expected temporal and tonal structures. When the stimulus properties conflict with internal expectancy, violations may register in deep primal structures, such as the amygdala and ventral striatum, that mediate emotion, arousal, motivation, and reward. The interpretive analysis of higher-order cognitive structures, including those involved in memory, on this integrative process and its consequent effects on limbic function could give rise to musical meaning. It is from this complex interaction of auditory, attentional, motor, emotion, and cognitive networks that feeling takes form and sound becomes music.

## Supporting Information

File S1 The Chopin expressive performance contains tempo and loudness variations as performed by an expert pianist.
(3.46 MB MP3)

File S2 The Chopin mechanical performance maintains constant tempo and sound intensity throughout the piece (equal to the mean tempo and sound intensity of the expressive performance).
(3.46 MB MP3)

File S3 Animation of the real-time changes in neural activity that were time-locked to the tempo fluctuations in a musical performance of Frédéric Chopin's Etude in E major, Op.10, No. 3. This animation includes a subset of the brain regions that exhibited time-locked activity. Shown are cortical and subcortical motor areas thought to be involved in pulse perception, and a network of areas consistent with the human ‘mirror neuron’ system. The specific brain regions are: Top left 2D rendering (top to bottom): right anterior cingulate, right basal ganglia (lentiform nucleus/putamen). Top middle 3D rendering (clockwise from top): bilateral supplementary motor area (SMA), primary motor cortex, left BA 44, right anterior cingulate. Bottom left 3D rendering (right hemisphere; moving counterclockwise from top): SMA and primary motor cortex, inferior parietal lobe BA 40, superior temporal sulcus, insula, ventral premotor cortex. Bottom right 3D rendering (left hemisphere, clockwise from top): SMA, primary motor cortex, BA 44, insula. Mean correlations (across listeners) were animated by weighting the tempo curve according to regional peak t-scores and projecting the result onto the corresponding brain areas.
(13 MB M4V ZIP)
